# Assessment of in silico protein sequence analysis in the clinical classification of variants in cancer risk genes

**DOI:** 10.1007/s12687-016-0289-x

**Published:** 2017-01-03

**Authors:** Iain D. Kerr, Hannah C. Cox, Kelsey Moyes, Brent Evans, Brianna C. Burdett, Aric van Kan, Heather McElroy, Paris J. Vail, Krystal L. Brown, Dechie B. Sumampong, Nicholas J. Monteferrante, Kennedy L. Hardman, Aaron Theisen, Erin Mundt, Richard J. Wenstrup, Julie M. Eggington

**Affiliations:** Myriad Genetic Laboratories, Inc., 320 Wakara Way, Salt Lake City, UT 84108 USA

**Keywords:** HBOC, Lynch syndrome, Sequence conservation, Missense mutation, Variant classification, Variants of uncertain significance

## Abstract

Missense variants represent a significant proportion of variants identified in clinical genetic testing. In the absence of strong clinical or functional evidence, the American College of Medical Genetics recommends that these findings be classified as variants of uncertain significance (VUS). VUSs may be reclassified to better inform patient care when new evidence is available. It is critical that the methods used for reclassification are robust in order to prevent inappropriate medical management strategies and unnecessary, life-altering surgeries. In an effort to provide evidence for classification, several in silico algorithms have been developed that attempt to predict the functional impact of missense variants through amino acid sequence conservation analysis. We report an analysis comparing internally derived, evidence-based classifications with the results obtained from six commonly used algorithms. We compiled a dataset of 1118 variants in *BRCA1*, *BRCA2*, *MLH1*, and *MSH2* previously classified by our laboratory’s evidence-based variant classification program. We compared internally derived classifications with those obtained from the following in silico tools: Align-GVGD, CONDEL, Grantham Analysis, MAPP-MMR, PolyPhen-2, and SIFT. Despite being based on similar underlying principles, all algorithms displayed marked divergence in accuracy, specificity, and sensitivity. Overall, accuracy ranged from 58.7 to 90.8% while the Matthews Correlation Coefficient ranged from 0.26–0.65. CONDEL, a weighted average of multiple algorithms, did not perform significantly better than its individual components evaluated here. These results suggest that the in silico algorithms evaluated here do not provide reliable evidence regarding the clinical significance of missense variants in genes associated with hereditary cancer.

**Electronic supplementary material:**

The online version of this article (doi:10.1007/s12687-016-0289-x) contains supplementary material, which is available to authorized users.

## Background

Accurate variant classification is of significant importance to patient care. In particular, clinically actionable variants have the potential to help guide medical management decisions. In such cases, these decisions are guided by the National Comprehensive Cancer Network (NCCN) guidelines (https://www.nccn.org/professionals/physician_gls/f_guidelines.asp). For example, due to the high risk of developing breast and/or ovarian cancer women with pathogenic variants in *BRCA1* and *BRCA2* are recommended to receive increased screening, chemoprevention, and prophylactic surgeries (Daly et al. 2016). Similarly, individuals with pathogenic variants in *MLH1* and *MSH2* are recommended to receive more frequent colonoscopies starting at an earlier age due to the risk of developing colorectal cancer (Provenzale et al. 2016).

Given the importance of accurate clinical variant classification, the American College of Medical Genetics (ACMG) have published guidelines for the interpretation of sequence variants that often require multiple lines of evidence (Richards et al. 2015). For some classes of variants, such as missense variants, obtaining this evidence can be a challenge. Missense variants result in amino acid substitutions in the protein product that may affect protein structure and/or function and, in rare cases, RNA splicing. However, commonly used criteria, including co-segregation analysis, population frequency data, *in-trans* observations with a deleterious mutation, and direct evidence of the functional impact, are often not available for these rare variants. As a result, in silico prediction tools are frequently used to evaluate the pathogenicity of missense variants (Pesaran et al. 2016; Thompson et al. 2014).

These computational tools are based on the premise that the evolutionary conservation of an amino acid in a protein reflects its absolute requirement for protein function. In hereditary cancer testing, several in silico tools are available to evaluate the pathogenicity of missense variants based on sequence conservation across multiple mammalian species (Ng and Henikoff 2001). Some algorithms also incorporate biochemical and/or biophysical parameters of the protein in order to improve their predictive accuracy (Adzhubei et al. 2010; Chao et al. 2008; Mathe et al. 2006; Tavtigian et al. 2006).

In silico tools were initially developed for academic research, where computational methods are often used to screen large amounts of data to identify candidate genes/variants for further investigation. With the increased use of clinical genetic testing and the challenges of classifying missense variants, in silico tools are now incorporated into professional society guidelines for variant classification (Easton et al. 2007; Plon et al. 2008; Richards et al. 2015) and are used for clinical variant classification. However, several studies have demonstrated that the specificity (correct identification of benign variants) of these tools can be as low as 13% (Chan et al. 2007; Doss and Sethumadhavan 2009; Flanagan et al. 2010; Gnad et al. 2013; Leong et al. 2015; Miosge et al. 2015; Ng and Henikoff 2002; Schiemann and Stowell 2016; Thusberg et al. 2011; Valdmanis et al. 2009) and with poor correlation between results from multiple programs (Thusberg et al. 2011). While these limitations may be acceptable in selecting variants for research, false positives and variability between methods may compromise patient care in a clinical setting.

Existing comprehensive reports on the clinical use of in silico tools have been limited to small cohorts (Hicks et al. 2011; Thompson et al. 2013) or variant datasets that are poorly curated (Thusberg and Vihinen 2009). In order to provide a comprehensive evaluation of their clinical use, we evaluated the predictive functionality of several in silico tools in a large cohort of individuals who underwent clinical genetic testing. Classifications for missense variants in *BRCA1*, *BRCA2*, *MLH1*, and *MSH2* that were based on independent lines of evidence were compared to classifications from six commonly used in silico tools: Align-GVGD, CONDEL, Grantham Analysis, MAPP-MMR, PolyPhen-2, and SIFT.

## Materials and methods

### Variant selection

All missense variants identified in *BRCA1*, *BRCA2*, *MLH1*, and *MSH2* by a commercial testing laboratory (Myriad Genetic Laboratories, Inc., Salt Lake City, UT) as of August 2013 were evaluated here. Variation in these high penetrance genes has the potential to significantly affect medical management. These variants were classified as pathogenic or benign based on multiple, independent lines of evidence that did not include sequence conservation (Eggington et al. 2013; Richards et al. 2015). This includes statistical methods (Pruss et al. 2014), biochemical/biophysical assays in the literature, population allele frequency thresholds, and co-occurrences with mutations in the same gene or pathway. Variants whose pathogenicity may arise from a mechanism not purely related to the amino acid substitution (variants that impact mRNA splicing, translation from the initiating 5′ AUG start codon, silent mutations) were excluded. This resulted in a final dataset of 1118 missense variants.

### In silico classification tools

The final set of variants was classified using the following commonly used in silico tools: SIFT, PolyPhen-2 (two available models: *HumDiv* and *HumVar*), Grantham matrix score, Align GVGD, MAPP-MMR, and CONDEL server. For Align-GVGD, an additional 316 *BRCA1* and 434 *BRCA2* variants were excluded from analysis, as they were used to train the algorithm and represent a possible source of statistical bias.

A brief summary of these algorithms is provided in Table 1 and the full details can be found in Online Resource 1. Briefly, these tools base variant classifications on sequence homology (SIFT, PholyPhen-2, MAPP-MMR, CONDEL), structural features (PolyPhen-2, CONDEL), and/or physiochemical properties (Grantham, Align-GVGD, MAPP-MMR). All in silico tools were applied to variants in *BRCA1*, *BRCA2*, *MLH1*, and *MSH2* with the exception of MAPP-MMR, which is only applicable for *MLH1* and *MSH2* variants. Each in silico classification tool utilizes different parameters for variant classification, the full details of which are in Online Resource 1.

**Table 1:** Description of in silico tools

In silico tool	Algorithm parameters	Notes
SIFT	• Sequence conservation	
PolyPhen-2	• Sequence conservation • Structure-based features	
Grantham matrix score	• Biochemical properties of amino acids	• Consideration of composition, polarity, and molecular volume of amino acids
Align-GVGD	• Sequence/biochemical variation	• Extension of Grantham matrix score to sequence alignments • Requires a large training set of variants with known classifications
MAPP-MMR	• Sequence conservation • Biochemical properties	• Only used for *MLH1* and *MSH2*
CONDEL server	• Sequence conservation • Structure-based features	• Weighted combination of SIFT, PolyPhen-2 and MutationAssessor scores

*SIFT* sorting intolerant from tolerant, *PolyPhen-2* polymorphism phenotyping v2, *MAPP-MMR* multivariate analysis of protein polymorphism, mismatch repair, *CONDEL server* CONsensus DELeteriousness score of missense SNVs

### Evaluation of in silico classification tool performance

For all genes and algorithms, we calculated the overall accuracy (ratio of overall correct predictions to the total number of predictions), specificity (correct identification of benign variants; true negative rate), and sensitivity (correct identification of pathogenic variants; true positive rate) relative to the testing laboratory classifications. Classifications were evaluated in three tiers (benign, uncertain, and pathogenic), and those of the same pathogenicity were considered concordant. For example, a laboratory classification of deleterious and an in silico classification of damaging would be considered concordant while a laboratory classification of benign and an in silico classification of unclassified would be considered discordant.

The positive predictive values (PPV, the proportion of positive results that were true positives) and negative predictive values (NPV, proportion of negative results that were true negatives) were also calculated.

As variant cohort size varied between algorithms, the Matthews correlation coefficient was used to provide a balanced comparison between in silico tools. This metric is not affected by sample size or the proportion of neutral to pathogenic variants in each dataset. A value of +1 indicates a perfect prediction while a value of −1 indicates a total disagreement between the in silico predicted classification and the laboratory classification. The full details of all statistical evaluations performed here can be found in Online Resource 1.

## Results

Overall, a maximum of 1118 variants were available for analysis. Table 1 shows the distribution of variants evaluated by gene and by in silico method. Aside from MAPP-MMR, which was only available for *MLH1* and *MSH2*, Align-GVGD had the lowest number of evaluable variants (*n* = 368). This was due to the large number of variants that were used to initially train the algorithm and that we eliminated from our analyses to mitigate possible statistical bias.

### Overall in silico algorithm performance

Table 2 shows the results of the in silico classification evaluation for the overall analysis, with the highest accuracy being observed for Align-GVGD (90.8%). This algorithm also showed the least divergence between specificity (91.7%) and sensitivity (84.1%). Still, approximately 1 in 10 patients would receive a false negative with this tool and about 1 in 5 would receive a false positive. The number of true negatives, true positives, false positives, and false negatives are provided in Online Resource 2. While the NPV for Align-GVGD was 97.7%, the PPV was only 57.8%.

**Table 2:** Performance of in silico tools

Algorithm	Total variants	Accuracy	Sensitivity	Specificity	PPV	NPV
Overall performance						
Align-GVGD	368	90.8%	84.1%	91.7%	57.8%	97.7%
SIFT	1118	60.2%	99.0%	56.4%	18.2%	99.8%
PolyPhen-2 *HumDiv*	1118	58.7%	90.0%	55.6%	16.6%	98.3%
PolyPhen-2 *HumVar*	1118	71.1%	81.0%	70.1%	21.0%	97.4%
CONDEL	1109	69.8%	84.4%	68.4%	20.2%	97.9%
Grantham	866	67.1%	78.2%	66.0%	18.5%	96.8%
MAPP-MMR^a^	71	74.6%	100.0%	56.1%	62.5%	100.0%
*BRCA1*						
Align-GVGD	103	97.1%	91.7%	97.8%	84.6%	98.9%
SIFT	419	48.9%	100.0%	41.5%	19.9%	100.0%
PolyPhen-2 *HumDiv*	419	55.6%	83.0%	51.6%	19.9%	95.5%
PolyPhen-2 *HumVar*	419	69.2%	67.9%	69.4%	24.3%	93.7%
CONDEL	414	67.4%	76.0%	66.2%	23.6%	95.3%
Grantham	329	69.0%	90.2%	66.0%	27.4%	97.9%
*BRCA2*						
Align-GVGD	165	92.1%	100.0%	92.1%	7.1%	100.0%
SIFT	599	60.9%	100.0%	59.9%	6.4%	100.0%
PolyPhen-2 *HumDiv*	599	73.6%	100.0%	72.9%	9.2%	100.0%
PolyPhen-2 *HumVar*	599	67.8%	100.0%	66.9%	7.7%	100.0%
CONDEL	596	71.8%	100.0%	71.0%	8.7%	100.0%
Grantham	455	64.4%	53.8%	64.7%	4.3%	97.9%
*MLH1*						
Align-GVGD	49	79.6%	76.2%	82.1%	76.2%	82.1%
SIFT	49	71.4%	100.0%	50.0%	60.0%	100.0%
PolyPhen-2 *HumDiv*	49	59.2%	95.2%	32.1%	51.3%	90.0%
PolyPhen-2 *HumVar*	49	67.3%	95.2%	46.4%	57.1%	92.9%
CONDEL	49	69.4%	90.5%	53.6%	59.4%	88.2%
Grantham	39	71.8%	68.8%	73.9%	64.7%	77.3%
MAPP-MMR	36	86.1%	100.0%	68.8%	80.0%	100.0%
*MLH2*						
Align-GVGD	51	84.3%	90.0%	82.9%	56.3%	97.1%
SIFT	51	52.9%	90.0%	43.9%	28.1%	94.7%
PolyPhen-2 *HumDiv*	51	56.9%	100.0%	46.3%	31.3%	100.0%
PolyPhen-2 *HumVar*	51	60.8%	90.0%	53.7%	32.1%	95.7%
CONDEL	50	66.0%	88.9%	61.0%	33.3%	96.2%
Grantham	43	76.7%	75.0%	77.1%	42.9%	93.1%
MAPP-MMR	35	62.9%	100.0%	48.0%	43.5%	100.0%

^a^Only used for *MLH1* and *MSH2*

SIFT, PolyPhen-2, CONDEL, and Grantham had overall accuracies ranging from 58.7% (PolyPhen-2 HumDiv) to 71.1% (PolyPhen-2 HumVar) (Table 2). Although the sensitivity of these algorithms ranged from 78.2% (Grantham) to as high as 99.0% (SIFT), the specificities were much lower, ranging from 55.6% (PolyPhen-2 HumDiv) to 70.1% (PolyPhen-2 HumVar). The performance of the CONDEL algorithm (69.8%), which is a weighted average of SIFT, PolyPhen-2, and MutationAssessor, was not superior to SIFT and PolyPhen-2 in isolation. In addition, the NPVs for all algorithms were >95%; however, the PPVs were substantially lower at 16–20% (Table 2). Both the low specificities and PPVs reflect the high frequency of false positives for these in silico tools.

MAPP-MMR, which is specific to *MLH1* and *MSH2*, had an overall accuracy of 74.6% (Table 2). There was a large discrepancy between the sensitivity (100.0%) and specificity (56.1%). As such, the PPV was 62.5%.

**Fig. 1:**
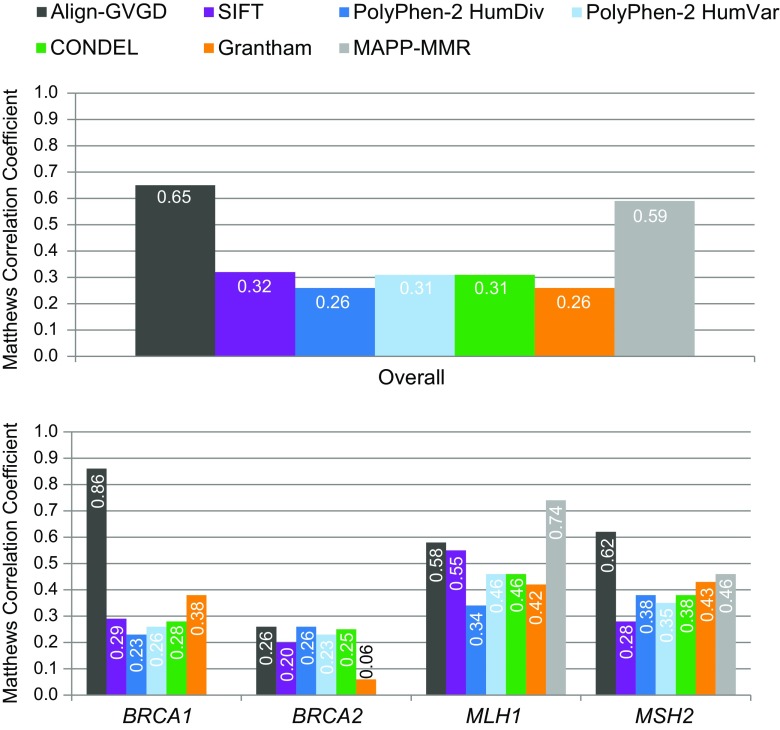
Overall (*top*) and gene-specific (*bottom*) Matthews correlation coefficients for in silico tools relative to laboratory classification

As the number of variants available for analysis for each in silico tool ranged from 71 (MAPP-MMR) to 1118 (SIFT, PolyPhen-2), we compared the performance of each algorithm in a manner that is independent of cohort size. Figure 1 (top) shows the overall Matthews correlation coefficients, where a value of 1 indicates complete concordance with the laboratory classifications. The highest degree of correlation was observed for Align-GVGD (0.65) then MAPP-MMR (0.59). The remaining in silico tools showed lower correlation with the laboratory classifications, which utilized multiple, independent lines of evidence, as detailed in the “Materials and methods,” with correlation coefficients ranging from 0.26 to 0.32.

### Classification of *BRCA1* and *BRCA2* variants

All algorithms except MAPP-MMR were used to classify missense variants in *BRCA1* and *BRCA2*. As observed for the overall algorithm performance, Align-GVGD had the highest overall accuracy for classification of *BRCA1* variants (97.1%) (Table 2). Again, the sensitivity and specificity were consistent, at 91.7 and 97.8%, respectively. As such, both the PPV (84.6%) and NPV (98.9%) were high. However, this same degree of predictive accuracy was not observed for the classification of missense variants in *BRCA2*. Although the overall accuracy, sensitivity, and specificity are relatively high, the PPV is only 7.1%. This means that there will be a very high frequency of false positives for *BRCA2* variants classified with Align-GVGD. This discrepant algorithm performance between *BRCA1* and *BRCA2* is also clear in Fig. 1, which shows the correlation between the algorithm classifications and the laboratory classifications. Despite a relatively high degree of correlation for *BRCA1* (0.86), the correlation coefficient is only 0.26 for Align-GVGD classification of *BRCA2* variants.

All other algorithms were poor predictors of the clinical classification of both *BRCA1* and *BRCA2* variants (Table 2). For *BRCA1* variant classification, overall accuracies ranged from 48.9% (SIFT) to 69.2% (PolyPhen-2 HumVar). Again, algorithm specificities (41.5–59.2%) and PPVs (19.9–27.4%) were low, which is representative of a large number of false positives (Online Resource 2). For example, *BRCA1* c.5317A>T (p. Thr1773Ser) was classified as benign by the reference laboratory based on published literature showing that the variant does not affect gene function (Lee et al. 2010) and phenotypic evidence from a family history weighting algorithm (Pruss et al. 2014) (Table 3). Although the variant was classified as benign by PolyPhen-2, it was classified as pathogenic by Align-GVGD and SIFT.

**Table 3:** Case examples of discrepant classifications

Laboratory classification	In silico classification(s)
*BRCA1* c.5317A>T (p. Thr1773Ser)	
Benign	Benign
• Phenotypic evidence based on family history weighting algorithm (Pruss et al. 2014) shows the variant is associated with less sever family history of *BRCA1*-associated cancers. • No folding defect in *BRCA1* protein; peptide binding activity normal; peptide binding specificity normal; transactivation activity uncertain (Lee et al. 2010).	• PolyPhen-2 (HumDiv and HumVar)
	Pathogenic
	• Align-GVGD, SIFT
*BRCA2* c.7994A>G (p. Asp2665Gly)	
Benign	Pathogenic/Likely Pathogenic
• *in trans* observation with *BRCA2* c.4398_4402del (p. Leu1466Phefs*2), which results in premature truncation of the BRCA2 protein at amino acid position 1467 and is classified as pathogenic. The patient lacked symptoms of the recessive phenotype associated with biallelic *BRCA2* mutations. • Phenotypic evidence based on family history weighting algorithm (Pruss et al. 2014) shows the variant is associated with less severe family history of *BRCA2*-associated cancers.	• Align-GVGD, PolyPhen-2 (HumDiv and HumVar), SIFT
*MLH1* c.394G>C (p. Asp132His)	
Benign	Pathogenic/Likely Pathogenic
• Phenotypic evidence based on family history weighting algorithm (Pruss et al. 2014) shows the variant is associated with less severe family history of *MLH1*-associated cancers. • *in trans* observation with the pathogenic *MLH1* variant, c.1731G>A (p. Ser577Ser), which is a silent variant that causes skipping of exon 15 (Pagenstecher et al. 2006) and has been described in multiple HNPCC families (Kohonen-Corish et al. 1996; Pagenstecher et al. 2006; Wu et al. 1997). The patient lacked symptoms of the recessive phenotype associated with biallelic *MLH1* mutations.	• Align-GVGD, PolyPhen-2 (HumDiv and HumVar), SIFT, MAPP-MMR

For *BRCA2* variant classification, SIFT, PolyPhen-2, CONDEL, and Grantham had overall accuracies ranging from 60.9 to 71.8%. Although nearly all algorithms had a sensitivity of 100%, the specificities ranged from 59.9% (SIFT) to 72.9% (PolyPhen-2 HumVar) (Table 2). Again, this was accompanied by extremely low PPVs (4.3–9.2%) due to a high frequency of false positives. For example, *BRCA2* c.7994A>G (p. Asp2665Gly) was classified as benign by the reference laboratory based on an *in-trans* observation (occurring in the same gene but different allele) with a known pathogenic *BRCA2* variant (Table 3). However, all four in silico tools that produced a classification for this variant called it either pathogenic or likely pathogenic.

The analysis in Fig. 1, which is independent of cohort size, shows that there was low correlation between the remaining algorithms and laboratory classifications. Most in silico tools had lower correlation coefficients for *BRCA2* classifications than for *BRCA1* classifications. This was most significant for Grantham, which dropped from 0.38 for *BRCA1* to 0.06 for *BRCA2*.

### Classification of *MLH1* and *MSH2* variants

The highest overall accuracy for *MLH1* missense variants was observed for MAPP-MMR (86.1%) (Table 2). However, the sensitivity and specificity were not consistent, at 100.0 and 68.8%, respectively. As a result, the NPV was high (100.0%) while the PPV was only 80.0%. Align-GVGD also had a relatively high overall predicative accuracy for the classification of *MLH1* variants at 79.6%. This algorithm also had similar sensitivity (76.2%) and specificity (82.1%). SIFT, PolyPhen-2, CONDEL, and Grantham had overall accuracies ranging from 59.2 to 71.8%. Algorithm specificities (32.1–73.9%) and PPVs (51.3–64.7%) were relatively low due to a high frequency of false positives (Online Resource 2). For example, *MLH1* c.394G>C (p. Asp132His) was classified as benign by the reference laboratory based on an *in-trans* observation with a known pathogenic variant and phenotypic evidence from a family history weighting algorithm (Pruss et al. 2014) (Table 3). However, this variant was classified as pathogenic or likely pathogenic by Align-GVGD, PolyPhen-2, SIFT, and MAPP-MMR.

For *MSH2* variant classification, the overall accuracy of MAPP-MMR dropped to 62.9%. Align-GVGD had the highest overall accuracy for *MSH2* variant classification, at 84.3%. SIFT, PolyPhen-2, CONDEL, and Grantham had lower overall accuracies, which ranged from 52.9 to 66.0%. Again, the specificity for nearly all algorithms was much lower than the sensitivity. As such, the PPVs ranged from 28.1% (SIFT) to 56.3% (Align-GVGD) (Table 2). This again shows that the use of in silico tools for *MSH2* missense variant classification results in a high incidence of false positives. Figure 1 shows that the correlation coefficients for *MLH1* were generally higher than those for *MSH2*; however, all algorithms showed relatively low degrees of correlation with the reference laboratory classifications, with the exception of MAPP-MMR for *MLH1* classification.

## Discussion

We assessed the predictive functionality of six commonly used in silico tools for the assessment of missense variants in a large dataset of *BRCA1*, *BRCA2*, *MLH1*, and *MSH2* variants identified during the course of clinical genetic testing. All algorithms in this study generated high error rates (high frequency of false positives, low correlation coefficients) when compared to the reference classifications. These results are consistent with previous studies that also reported a high rate of error from in silico-derived classifications (Flanagan et al. 2010; Gnad et al. 2013; Hicks et al. 2011; Martelotto et al. 2014; Miosge et al. 2015; Thusberg et al. 2011).

### Sources of error for in silico tools

There are several sources of error that may contribute to the inconsistent and unreliable performance of in silico tools. First is the need for accurate sequence alignments in order for the algorithm to accurately assess conservation. As this process is essentially heuristic, the accuracy of the alignment and the predicted result falls off dramatically in poorly conserved regions of the protein. As demonstrated by Align-GVGD, sequence alignments may be corrected by incorporating information from available protein structures; however, it is likely that many homologous structures would be required to sufficiently improve the accuracy for clinical use. It would be more appropriate to perform a full structural analysis, which would also provide direct evidence regarding the effect of the variant on protein structure and function. While attempts may be made to manually curate sequence alignments, this can lead to misaligned regions of the proteins in the absence of additional information to help guide the process. For this reason, manual sequence conservation analysis should be avoided as it is likely to incorporate even more uncertainty.

The second source of error is the assumption that a conserved position in multiple sequence alignments is more important for protein function than one that is less well conserved. As recently alluded to by MacArthur et al., this hypothesis is limited by a lack of proper biological context (MacArthur et al. 2014). Pathogenic variants can be observed at sites of relatively low conservation while benign variants may be found in areas of the protein sequence that are highly conserved throughout mammalian evolution (Hicks et al. 2011).

### Challenges with training in silico tools

The poor overall predictive accuracy of the in silico tools assessed here may be related to the challenges in “training” the algorithms. Given the rarity of individual missense variants and the resulting limited available evidence of pathogenicity, it is challenging to obtain enough robustly classified variants to both train and independently evaluate the algorithm. When these two datasets overlap, the data can be “overfit” and the reported predictive accuracy of the tool is often artificially inflated. A recent study by Grimm et al. highlighted that accurate predictions from in silico tools are less frequently observed for variants which overlap between the training dataset and the evaluation dataset (Grimm et al. 2015). This overfitting may also occur when predictions from tools are combined (i.e., CONDEL).

For Align-GVGD, we were able to selectively evaluate the algorithm on a curated set of variants, to maximize the overall predictive accuracy. As such, while this in silico tool had the best overall performance, it is unlikely that this accuracy could be replicated outside of the current study. Additionally, this approach is not feasible for genes in which few discrete missense variants have been identified and robustly, clinically classified.

The exclusion of a large number of *BRCA1* and *BRCA2* variants that were used to train Align-GVGD resulted in the smallest cohort size in this study. In the case of *BRCA1*, this may have affected the specificity, which is seemingly the highest across all genes studied using any of the six in silico tools employed. However, the PPV indicates that the ratio of true positives (TP) to the total number of positives (TP + FP) in the *BRCA1* dataset was still only 84.6% for Align-GVGD.

For the remaining algorithms, we were unable to avoid any potential overfitting, instead relying on the built-in algorithm training. As such, it is not surprising that the performance of these in silico tools in this study was much lower than previously reported. The low PPVs observed for all algorithms across all genes investigated here indicate that there is a high frequency of false positive results when using in silico tools for the classification of variants in cancer-risk genes. This is exemplified by the three case examples given in Table 3, where independent evidence of pathogenicity including published literature and *in-trans* observations with a pathogenic variant resulted in a benign classification. In the absence of additional evidence, these false positives may result in inappropriate clinical management. This is especially troubling for *BRCA1* and *BRCA2*, as pathogenic variants in these genes are potential indications for prophylactic surgeries (Daly et al. 2016).

### Academic vs clinical utility

Establishing the relationship between genotype and phenotype is a fundamental principle of genetics. In an academic research laboratory, biological samples and corresponding phenotypic data are used to gain knowledge of this relationship. Given the size of the human genome, spectrum of variation, and ever decreasing costs of genotyping, researchers may be left with large volumes of candidate variants after association analysis. In such situations, in silico prediction tools are useful for contextualizing and prioritizing candidate variants for further follow-up, especially when functional experimental assays are not readily available or are cost prohibitive. One of the consequences of using these tools is that relevant data points may be discarded. However, the knowledge gained from these studies is often used to guide further research and not direct the medical management of a patient.

In a clinical laboratory, samples are tested due to clinical suspicion of hereditary cancer risk. Accurate variant classification using multiple lines of evidence is vital for appropriate clinical management based on NCCN guidelines. In this setting, incorrect variant classification based on in silico tools comes with more immediate consequences to the patient.

### Additional considerations for clinical use

We have outlined many challenges associated with the use of several specific in silico tools in the clinical setting. As discussed previously, this includes the requirement of large training sets, unclear variant classification, and inaccurate biological assumptions. Caution must also be used when sequence conservation information is included in multifactorial models to establish posterior probability. Such methods calculate a prior probability of causality (calculated from in silico sequence conservation), which is then combined with various likelihoods of causality including co-segregation analysis, *in-trans* observations, family history, and histopathology data to give a final probability of causality for a variant (Lindor et al. 2013; Lindor et al. 2012).

The inherent problem with these methods is that the separate probabilities and likelihoods are combined (i.e., the various lines of evidence are never truly considered “independent” of one another). The analysis is therefore weighted by the prior sequence conservation analysis, which, as we have shown here, is a poor predictor of the clinical significance of a variant. In addition, any thresholds for pathogenicity used in such a multifactorial analysis would have to be thoroughly clinically validated.

## Conclusions

Classification of missense variants can be a challenging process, particularly for clinical use. Prediction of their possible cancer association is especially difficult in pleiotropic genes such as *BRCA1*, where disruption of a specific function or domain may not necessarily affect downstream tumor suppression activity (Shakya et al. 2011). One must therefore be careful to ensure that a loss of function associated with a missense variant correlates with increased predisposition to a given cancer syndrome. In general, the shortcomings of in silico sequence conservation analysis may be described as applying a one- or two-dimensional solution to a much more complex, three-dimensional problem. While some algorithms may incorporate biochemical features or limited structural analysis, these additions were insufficient to overcome the inherent low accuracy in these methods for variant classification in the cancer-risk genes evaluated here. Although a full structural analysis is far more appropriate, this must be carefully undertaken by a trained structural biologist.

The low specificity of these methods is of particular concern given the potential for more aggressive medical management strategies including prophylactic surgeries following a false-positive result. Since the primary aim of clinical testing is to provide results to inform medical management, variant classification techniques must reach an exceptionally high level of confidence compared to academic research use. For the purposes of purely academic research, the rates of false-positives and false-negatives are less problematic as long as the results are not applied to patient care. However, to confidently inform a clinical decision, the thresholds for accuracy need to be exceedingly high, well above the average sensitivity/specificity seen in this study (Akobeng 2008). We therefore conclude from this analysis that the error rates of commonly used in silico tools are too high to warrant use as primary evidence to further assess variants of unknown significance in a clinical setting.

## Electronic supplementary material

Online Resource 1 Supplemental Methods detailing the in silico tools evaluated here, variant classification analysis, and statistical methods. (DOCX 36 kb)

Online Resource 2 Supplemental Table 1 showing the number of variants evaluated, true positive (TP), false positive (FP), false negatives (FN), and true negatives (TN) for each in silico tool overall and by gene. (DOCX 21 kb)
