# Supplementary material for: Assessment of in silico protein sequence analysis in the clinical classification of variants in cancer risk genes
Source: J Community Genet. 2017 Jan 3;8(2):87–95. doi: 10.1007/s12687-016-0289-x (PMC5386911; doi:10.1007/s12687-016-0289-x)
Supplement: Supplementary file 1 — Supplemental Methods detailing the in silico tools evaluated here, variant classification analysis, and statistical methods. (DOCX 36 kb) [file 12687_2016_289_MOESM1_ESM.docx]

**Assessment of sequence conservation analysis in clinical variant classification**

**Supplemental Methods**

***in silico Classification Tools***

**SIFT (Sorting Intolerant From Tolerant):** This tool uses sequence homology to predict whether a substitution affects protein function (http://sift.jcvi.org/). Variants are classified as “tolerated” or “deleterious” based on the conservation of the wild-type amino acid in MSAs constructed from closely related species.

**PolyPhen-2 (Polymorphism Phenotyping v2):** This tool uses a combination of sequence-based and structure-based features to predict the effect of a missense mutation on the structure and function of a protein (‘probably damaging’, ‘possibly damaging’, or ‘benign’) (http://genetics.bwh.harvard.edu/pph2/). For the purposes of this analysis, both ‘probably’ and ‘possibly’ damaging variants were treated as deleterious. We compared both models used to train the algorithm, *HumDiv* and *HumVar*.

**Grantham matrix score:** This tool reflects the difference between the wild-type and variant based on three physiochemical properties: composition (c), polarity (p) and molecular volume (v) ([Grantham 1974](#_ENREF_19)).

**Align-GVGD:** This tool extends the original Grantham concept to sequence alignments (http://agvgd.iarc.fr/agvgd_input.php). The algorithm uses two conservation scores: Grantham Variation (GV), a measure of the observed range of variation at a particular position in the alignment and the Grantham Deviation (GD), which measures the distance between a variant and GV at that position. Align-GVGD classifies missense mutations in genes of interest as ‘neutral’ (benign), ‘deleterious’ or ‘unclassified’ (VUS). Unclassified variants were excluded from further calculations.

**MAPP-MMR** **(Multivariate Analysis of Protein Polymorphism, Mismatch Repair):** This tool is a customized version of the MAPP algorithm, optimized for *MLH1* and *MSH2* (<http://mappmmr.blueankh.com/Impact.php>) ([Stone and Sidow 2005](#_ENREF_32)). The algorithm uses sequence conservation and a matrix of amino acid biochemical properties to estimate constraints that can be applied to each position in the alignment. The degree to which these constraints are violated by a variant at a given position is measured by the impact score.

**CONDEL server** (**CONsensus DELeteriousness score of missense SNVs):** This tool scores variants as neutral or deleterious based on a weighted average of scores (WAS) obtained from SIFT, PolyPhen-2 and MutationAssessor (http://bg.upf.edu/condel/analysis) ([Vihinen 2012](#_ENREF_37)).

***Variant Analysis by in silico Classification Tools***

Analyses with CONDEL, MAPP-MMR and SIFT (SIFT Human Protein DB) were all performed using the default parameters for each algorithm. We treated a MAPP-MMR score of 3.0–5.0 as borderline; variants above and below these thresholds were classified as deleterious or benign, respectively ([Chao et al. 2008](#_ENREF_6)). Borderline variants were treated as VUSs and excluded from further calculations. For Align-GVGD, the deepest alignments (i.e. those with the largest number of related sequences) gave the highest specificity and sensitivity. The results therefore represent the analysis based on the maximum alignment depth for each gene. Variants are scored by Align-GVGD using a 7-tiered class system (C0, C15, C25, C35, C45, C55 and C65). For the purpose of this analysis, Class C0 to C25 were scored as benign variants and Class ≥ C35 were scored as deleterious.

**Statistical analyses**

Each algorithm was assessed using the following statistical indicators:

$$Accuracy=\frac{TN+TP}{TP+TN+FP+FN}\text{x}100$$

$$Specificity=\frac{TN}{TN+FP}\text{x }100$$

$$Sensitivity=\frac{TP}{TP+FN}\text{x }100$$

$$PPV=\frac{TP}{TP+FP}\text{x}100$$

$$NPV=\frac{TN}{TN+FN}\text{x}100$$

For all reported statistical metrics, TN are true negatives, FN are false negatives, TP are true positives and FP are false positives, in comparison with reference classifications.

We also calculated the PPV (or ‘precision’, the proportion of positive results that were true positives), the NPV (proportion of negative results that were true negatives) and the Matthews correlation coefficient (MCC), using Myriad internal classifications as a reference. Unlike the other indicators, the MCC is unaffected by sample size and the proportion of neutral to pathogenic variants in a dataset, allowing for a more balanced comparison across datasets ([Baldi et al. 2000](#_ENREF_3); [Matthews 1975](#_ENREF_27); [Vihinen 2012](#_ENREF_39)). An MCC of +1 indicates a perfect prediction, 0 a random prediction and -1 a total disagreement between the predicted and the reference classification.
