# Supplementary material for: Assessment of in silico protein sequence analysis in the clinical classification of variants in cancer risk genes
Source: J Community Genet. 2017 Jan 3;8(2):87–95. doi: 10.1007/s12687-016-0289-x (PMC5386911; doi:10.1007/s12687-016-0289-x)
Supplement: Supplementary file 2 — Supplemental Table 1 showing the number of variants evaluated, true positive (TP), false positive (FP), false negatives (FN), and true negatives (TN) for each in silico tool overall and by gene. (DOCX 21 kb) [file 12687_2016_289_MOESM2_ESM.docx]

**Assessment of sequence conservation analysis in clinical variant classification**

**Supplemental Table 1.** Number of variants evaluated, true positive (TP), false positive (FP), false negatives (FN), and true negatives (TN) for each *in silico* tool overall and by gene.

| **Algorithm** | **Total Variants** | **TP** | **FP** | **FN** | **TN** |
| --- | --- | --- | --- | --- | --- |
| **Overall Performance** | | | | | |
| Align-GVGD | 368 | 37 | 27 | 7 | 297 |
| SIFT | 1118 | 99 | 444 | 1 | 574 |
| PolyPhen-2 *HumDiv* | 1118 | 90 | 452 | 10 | 566 |
| PolyPhen-2 *HumVar* | 1118 | 81 | 304 | 19 | 714 |
| CONDEL | 1109 | 81 | 320 | 15 | 693 |
| Grantham | 866 | 61 | 268 | 17 | 520 |
| MAPP-MMR* | 71 | 30 | 18 | 0 | 23 |
| ***BRCA1*** | | | | | |
| Align-GVGD | 103 | 11 | 2 | 1 | 89 |
| SIFT | 419 | 53 | 214 | 0 | 152 |
| PolyPhen-2 *HumDiv* | 419 | 44 | 177 | 9 | 189 |
| PolyPhen-2 *HumVar* | 419 | 36 | 112 | 17 | 254 |
| CONDEL | 414 | 38 | 123 | 12 | 241 |
| Grantham | 329 | 37 | 98 | 4 | 190 |
| ***BRCA2*** | | | | | |
| Align-GVGD | 165 | 1 | 13 | 0 | 151 |
| SIFT | 599 | 16 | 234 | 0 | 349 |
| PolyPhen-2 *HumDiv* | 599 | 16 | 158 | 0 | 425 |
| PolyPhen-2 *HumVar* | 599 | 16 | 193 | 0 | 390 |
| CONDEL | 596 | 16 | 168 | 0 | 412 |
| Grantham | 455 | 7 | 156 | 6 | 286 |
| ***MLH1*** | | | | | |
| Align-GVGD | 49 | 16 | 5 | 5 | 23 |
| SIFT | 49 | 21 | 14 | 0 | 14 |
| PolyPhen-2 *HumDiv* | 49 | 20 | 19 | 1 | 9 |
| PolyPhen-2 *HumVar* | 49 | 20 | 15 | 1 | 13 |
| CONDEL | 49 | 19 | 13 | 2 | 15 |
| Grantham | 39 | 11 | 6 | 5 | 17 |
| MAPP-MMR | 36 | 20 | 5 | 0 | 11 |
| ***MLH2*** | | | | | |
| Align-GVGD | 51 | 9 | 7 | 1 | 34 |
| SIFT | 51 | 9 | 23 | 1 | 18 |
| PolyPhen-2 *HumDiv* | 51 | 10 | 22 | 0 | 19 |
| PolyPhen-2 *HumVar* | 51 | 9 | 19 | 1 | 22 |
| CONDEL | 50 | 8 | 16 | 1 | 25 |
| Grantham | 43 | 6 | 8 | 2 | 27 |
| MAPP-MMR | 35 | 10 | 13 | 0 | 12 |
